# Functionalized MWCNTs-quartzite nanocomposite coated with *Dacryodes edulis* stem bark extract for the attenuation of hexavalent chromium

**DOI:** 10.1038/s41598-021-92266-0

**Published:** 2021-06-16

**Authors:** James F. Amaku, Segun Ogundare, Kovo G. Akpomie, Collins U. Ibeji, Jeanet Conradie

**Affiliations:** 1 Department of Chemistry, Michael Okpara University of Agriculture, Umudike, Nigeria; 2 Chemical Sciences Department, Olabisi Onabanjo University, Ago-Iwoye, Nigeria; 3 Department of Chemistry, University of the Free State, Bloemfontein, South Africa; 4 Department of Pure & Industrial Chemistry, University of Nigeria, Nsukka, Nigeria

**Keywords:** Ecology, Limnology, Chemistry, Engineering, Materials science

## Abstract

Multiwalled carbon nanotubes/quartzite nanocomposite modified with the extract of *Dacryodes edulis* leaves was synthesized and designated as Q, which was applied for the removal of Cr(VI) from water. The adsorbents (PQ and Q) were characterized using the SEM, EDX, FTIR, TGA, XRD, and BET analyses. The XRD revealed the crystalline composition of the nanocomposite while the TGA indicated the incorporated extract as the primary component that degraded with an increase in temperature. The implication of the modifier was noticed to enhance the adsorption capacity of Q for Cr(VI) by the introduction of chemical functional groups. Optimum Cr(VI) removal was noticed at a pH of 2.0, adsorbent dose (50 mg), initial concentration (100 mg dm^−3^), and contact time (180 min). The kinetic adsorption data for both adsorbents was noticed to fit well to the pseudo-second-order model. The adsorption equilibrium data were best described by the Langmuir model. The uptake of Cr(VI) onto PQ and Q was feasible, endothermic (ΔH: PQ = 1.194 kJ mol^−1^ and Q = 34.64 kJ mol^−1^) and entropy-driven (ΔS : PQ = 64.89 J K^−1^ mol^−1^ and q = 189.7 J K^−1^ mol^−1^). Hence, the nanocomposite demonstrated potential for robust capacity to trap Cr(VI) from aqueous solution.

## Introduction

The volume of waste generated and discharged into the water bodies has become a potential threat to man, marine, and the ecosystem. An increase in water pollution has declined the sources of potable water supply, hence, severe global water scarcity is foreseen in the nearest future. Natural disasters and anthropogenic activities are the major sources of water contaminants. These contaminants are mostly organic and inorganic^1^. Inorganic contaminants such as heavy metals are recalcitrant and often observed to be the major toxic materials in rivers, lakes, and groundwater. This could be due to the fact that they are non-degradable, bioaccumulate, and biomagnified^2–3^. Among these heavy metals, chromium in its highest oxidation state (Cr(VI)) is found to be notoriously toxic with a strong propensity to induce cancer, which could be associated with its high solubility, oxidative tendency, and ability to interact with the DNA^4^. Long-term exposure to Cr(VI) may also result in skin diseases, nose bleeding, respiratory tract infections, compromised immune system, hepatic diseases, genotoxic defect, and mutation^5–9^.

In an attempt to establish chromium-free water bodies, researchers have applied various physicochemical procedures to remove Cr(VI) from the loaded wastewater. Some of these methods include adsorption^10–11^, solvent extraction^9,12–13^, evaporation^14^, ion exchange^15^, and reduction^16^ amongst others. With exception to adsorption, some of the aforementioned procedures are either very expensive to operate or ineffective at low analyte concentration. However, the adsorption technique has demonstrated a good economic edge over the others and a great capacity to effectively eliminate Cr(VI) from the aqueous phase^17^. In this procedure, the synergistic reduction-adsorption mechanism amongst others has attracted great attention as it reduces hazardous Cr(VI) to a less toxic ion (Cr(III)). When Cr(VI) comes in contact with an adsorbent that acts as a reducing agent, it is readily being reduced to Cr(III) in an acidic medium due to its high redox potential value. Concerning the reduction-adsorption approach, adsorbents such as activated carbon^18–19^, rice bran^20^, graphene^21–22^, carbon nanotubes^23–25^, shale^26^, biomass^27–29^, nano-zerovalent metallic particles^30–32^, dolerite^33^, seeds^34^, chitosan^35–37^ amongst others have been modified due to their inherent limitation such as poor functionality that has hindered their application in environmental remediation practice. Hence, it is imperative to design a novel adsorbent that is not just effective but also green and scalable for the removal of Cr(VI) in a large volume of wastewater.

The application of carbon nanotubes (CNTs) in the various field could be attributed to their chemical^38^, optical^39^, mechanical^40^, thermal^41^, and electrical properties^42^. Similarly, the large surface areas, density, and porosity of CNTs project their application as adsorbents for the adsorption of both organic and inorganic pollutants. The surface modification of CNTs via acid treatment leads to the introduction of oxygen functionality such as hydroxyl (-OH) and carboxylic acid (-COOH) functional groups^43–46^. The introduced oxygen functionality often enhances the capacity of CNTs to trap pollutants of different chemistry from wastewater^47–50^. Hence, nanocomposites designed from CNTs may have a dynamic property fit for the removal of Cr(VI) from an aqueous system.

The metamorphic rock quartzite is known for its exceptionally hard, impermeable, and vitreous luster characteristic. This rock is rich in quartz and contains less than 10% of other minerals^51^. Quartzites are generally used as countertops in the kitchen, tiles, and in some cases as material for road construction. The application of this material as an adsorbent may be beneficially owing to its thermal stability and resistance to physical and chemical weathering under different pH conditions. Besides, quartzites are readily available and comparatively cheap. Hence, green surface modification of quartzite rock would present this rock as a value-added product for environmental remediation practices.

*Dacryodes edulis* also known as ‘African plum’, ‘native pear’, “African pear” or ‘bush butter’ belongs to the *Burseraceae* family. Different parts of this plant have served as a source of food and medicine to man^52–53^. Meanwhile, *Dacryodes edulis* leaf extract has demonstrated good ferric reducing antioxidant power (FRAP)^54^. Hence, the application of *Dacryodes edulis* leaves extract as modifier may be another path to exhaustively utilize the reducing potential of this tree plant. In this case, the plant extract may function as a reductant of the metal, initiating the attraction of Cr(VI) in wastewater for effective and rapid adsorption. Hence, this study aims to fabricate a nanocomposite via the green synthetic route by making use of quartzites rock, multiwalled carbon nanotube (MWCNTs), and *Dacryodes edulis* leaves extract for the adsorption of Cr(VI) from wastewater.

## Materials and methods

### Chemicals

Potassium dichromate (K_2_Cr_2_O_7_), sodium hydroxide (NaOH), sulphuric acid (H_2_SO_4_), hydrochloric acid (HCl), nitric acid (HNO_3_), sodium chloride (NaCl), and 1,5-diphenylcarbazide (DPC) were purchased from Sigma-Aldrich and used without further treatment. Similarly, multi-walled carbon nanotubes (MWCNTs) were procured from Sigma-Aldrich and purified before application.

### Samples and sample preparation

The leaves of *Dacryodes edulis* (DEL) were collected as waste from Umueze Umunumo (5° 40′ 13ʺ North, 7° 16′ 55ʺ East) in Ehime Mbano Local Government Area of Imo State, Nigeria. Thereafter, the leaves were deposited at the Department of Forestry, Micheal Okpara University of Agriculture Umudike. The leaves were identified by Mr Ibe M, and declared not endangered. The plant sample was cleaned using double distilled water. The clean DEL was air-dried and reduced to a fine powder by making use of an electric grinder. The pulverized DEL was then stored in an airtight container. About 300 g of powered DEL was extracted using double distilled water (0.5 dm^3^) at room temperature for 7 days. Thereafter, the extract was concentrated and stored for future application.

### Quartzite rock preparation

Quartzite rock was collected from the Department of Geology, Michael Okpara University of Agriculture Umudike, Abia State, Nigeria. The rock was washed using deionized water, dried, and thereafter crushed to a fine powder using a ball mill machine. About 15 g of the crushed quartzite was transferred into a 500 cm^3^ beaker containing 200 cm^3^ of 0.1 M HCl. The mixture was stirred for 3 h, filtered, and washed under vacuum using deionized water and designated as PQ.

### Acid treatment of MWCNTs

About 1 g of MWCNTs was treated with 50 cm^3^ of 6 M HCl for 3 h on stirring, diluted, and filtered. Thereafter, the HCl treated MWCNTs was further purified by making use of 50 cm^3^ of 6 M HNO_3_, using a similar experimental procedure as for HCl. The black product obtained was then dried and stored for further treatment. To functionalize the nanotubes, about 1 g of purified MWCNTs was weighed into a 250 cm^3^ beaker containing 100 cm^3^ of an acid mixture (nitric acid-sulfuric 3:1 (v/v)). The mixture was stirred for 12 h, diluted, filtered, washed to neutral using deionized water, dried, and stored for further application.

### Preparation of nanocomposite

Two grams of the functionalized multiwalled carbon nanotubes (*f-*MWCNTs) was weighed into a beaker containing 6 g of PQ and 20 cm^3^ of deionized water, the mixture was stirred for 6 h. Thereafter, 5 cm^3^ of glutaraldehyde was added to the mixture (*f-*MWCNTs/PQ). The mixture was then stirred to dryness at 110 °C. About 5 g of the black product obtained (crosslinked *f-*MWCNTs/PQ) was transferred into 50 cm^3^ of concentrated *Dacryodes edulis* leave aqueous extract. The mixture was stirred to dryness at 80 °C, vacuum oven-dried, ground, and stored in an airtight container and designated as Q.

### Characterization

The adsorbents (PQ and Q) were fabricated and characterized by making use of powder X-ray diffraction (XRD Bruker D8 Advance powder x-ray diffraction, Bruker, USA). The behaviour of Q in a thermally varied environment was investigated by making use of the PerkinElmer si-multaneous thermal analyzer STA6000 instrument, USA. Further characterization involved the use of Fourier transform infrared (FTIR) spectroscopy (Thermo Nicolet-870 spectrophotometer, USA), and scanning electron microscopy (ZEISS ultra plus, USA) coupled with the energy dispersive X-ray (EDX) spectroscopy. The specific surface area, pore-volume, and size of PQ and Q were assessed using the Brunauer–Emmett–teller (BET) nitrogen sorption–desorption method (Micromeritics Instruments Corp., USA).

### Determination of pH point of zero charge (pH_***PZC***_)

To determine the pH at the point of zero charges of PQ and Q, about 0.1 g of the materials was transferred into eleven 250 cm^3^ glass Erlenmeyer flasks with each containing 50 cm^3^ of 0.1 mol dm^−3^ NaCl solution at pH values ranging from 2 to 12. The flasks were stoppered and agitated for 48 h in a preset thermo-regulated water bath at 25 °C. The final pH of the mixture was obtained after 48 h and a plot of the final pH versus the initial pH was made from which the pH_PZC_ of PQ and Q was extrapolated from the line intercept^55^.

### Batch adsorption experiments

Batch adsorption experiments were performed by contacting a specific amount of adsorbent and 25 cm^3^ of Cr(VI) solution in a 100 cm^3^ stoppered glass bottle fixed on a preset thermostatic shaker. The implication of contact time, adsorbent dose, initial Cr(VI) concentration, solution pH, and temperature of the Cr(VI) solutions were assessed. The determination of optimum adsorption time was achieved by contacting the adsorbents for a specific time interval (5 to 1440 min). The conditions for the contact time experiment include 50 mg of adsorbent dose and 25 cm^3^ of 100 mg dm^−3^ Cr(VI) solution. To investigate the adsorbent dose experiments, 10–400 mg of adsorbents was employed. For the determination of the effect of solution pH, 100 mg dm^−3^ Cr(VI) solution was prepared and adjusted to a range pH 1 to 12 using 0.1 mol dm^−3^ H_2_SO_4_ or 0.1 mol dm^−3^ NaOH and each was agitated with 0.05 g of PQ or Q for 24 h. A concentration range of 10 to 100 mg dm^−3^ was chosen for initial Cr(VI) concentration experiments, 25 cm^3^ of the sorbate was adjusted to predetermined pH and contacted with 0.05 g either of the sorbents for 24 h under constant shaking at 298 K. To assess the thermodynamics of the adsorptive process, the initial concentration experiment was repeated for 303 K, 308 K and 313 K. The residual concentration of Cr(VI) was estimated by the colorimetric method using 1,5-diphenyl-carbazide as a complexing agent, analyzed using the UV–visible spectrophotometer at 540 nm^56^. The adsorption capacities and the uptake efficiency of PQ and Q were calculated as shown in the supplementary information.

### Kinetics and isotherm models

The kinetic modeling of adsorption was performed using four kinetic models while the isotherm analysis was carried out by applying eight isotherm models as described in the supplementary information.

### Reusability experiments

To examine the reusability of PQ and Q, the adsorbents were used to remove the adsorbate (Cr(VI)) from the aqueous phase using the same adsorption procedure stated in the previous section. The regeneration of PQ-Cr and Q-Cr was performed by making use of NaOH. About 0.5 g of PQ-Cr or Q-Cr was in contact with 25 cm^3^ of 0.5 mol dm^−3^ NaOH for 3 h at 25 °C. The regenerated adsorbents were washed twice with ultrapure water and dried for the next cycle. The removal efficiency of PQ and Q for the next cycle was estimated using Eq. (2).

### Compliance with ethical standard

The collection of plant materials complied with relevant institutional, national, international guidelines and legislations.

## Results and discussion

### Characterizations of adsorbents

**Figure 1:**
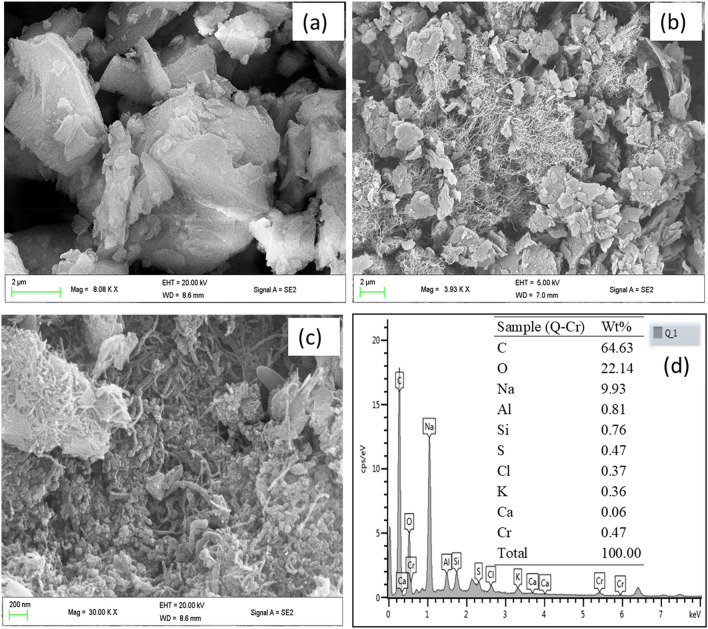
The SEM micrographs of (**a**) PQ, (**b**) Q, (**c**) Q-Cr and the EDX spectrum of Q-Cr (inset: Table of the elemental composition of the spot scanned).

The micrographs of the surface morphology of PQ and Q are displayed in Fig. 1. The SEM micrograph of the pristine quartzite (PQ) revealed an aggregate of smooth particles with varied shapes and sizes (Fig. 1a). The structure of PQ reflected the metamorphic nature of quartzite rock. The micrograph (Fig. 1b) of the nanocomposite(Q) showed that the quartzite particles were rapped with an intertwined network of cylindrical tubes resulting in the formation of microscopic channels on the surface with an increase in surface roughness probably due to coating by the incorporated extract. The micrograph (Fig. 1c) of the nanocomposite adsorbed with Cr(VI) (Q-Cr) was acquired at a higher magnification which further revealed an increase in surface roughness of the grains without the segregation of the network of carbon nanotubes or comparable sign of degradation concerning the surface integrity of the nanocomposite. This indicated the stability of the material in the aqueous environment. To further confirm the adsorption of chromium onto the surface of Q, the acquired EDX of Q-Cr (Fig. 1d) revealed the surface adsorbed chromium. It should be noted that the percentage (0.47%) indicated by EDX represented Cr on the spot being scanned and not the total adsorbed Cr on the nanocomposite.

**Figure 2:**
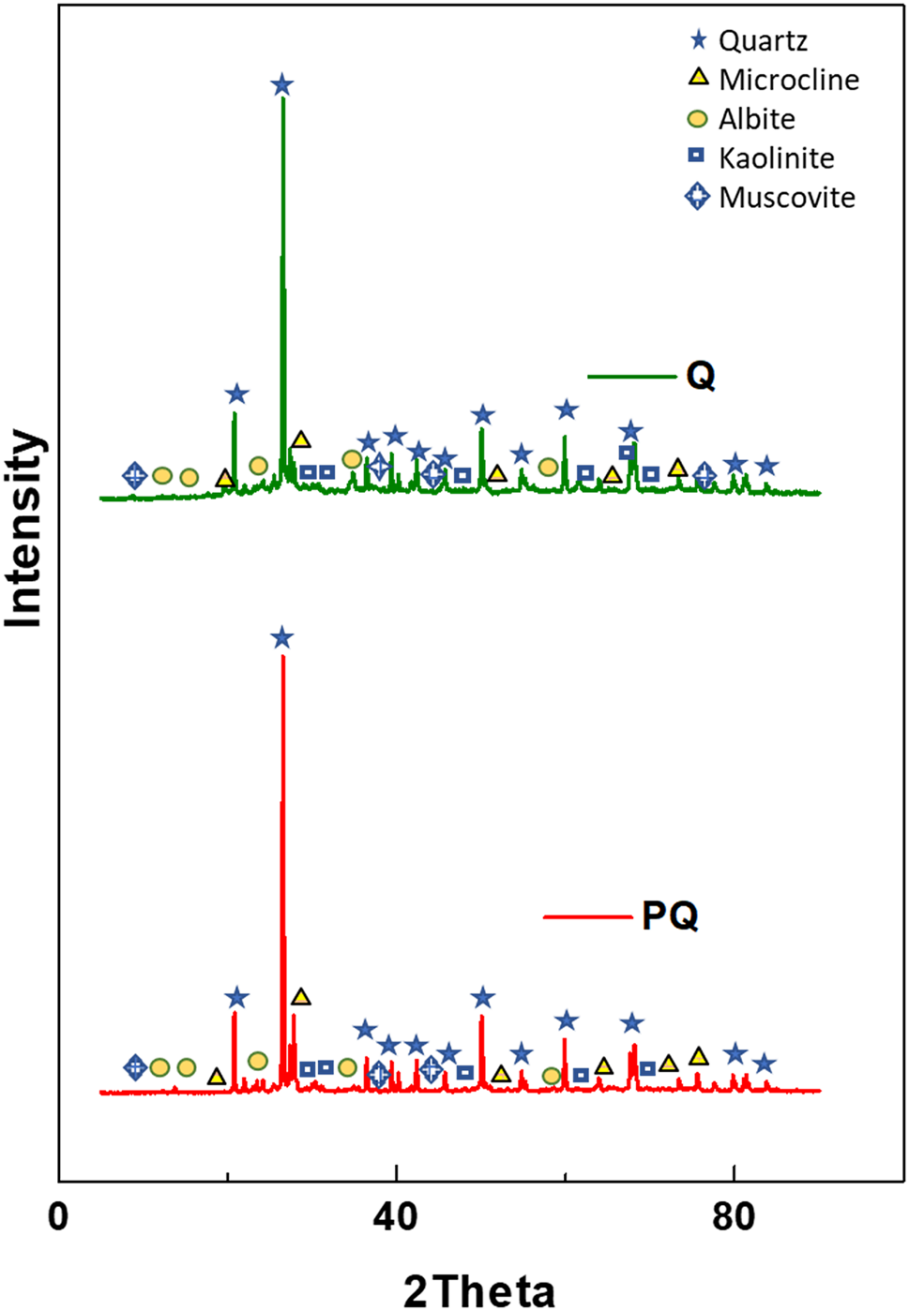
X-ray diffraction spectral for PQ and Q.

X-ray diffraction analysis was used to assess the mineral content of quartzite. As shown in Fig. 2, the pristine quartzite (PQ) contained quartz (SiO_2_, 72.07%), microcline (KAlSi_3_O_8_, 11.67%), albite (NaAlSi_3_O_8_, 4.17%), kaolinite (Al_2_Si_2_O_5_(OH)_4_, 0.69%), and muscovite (KAlSi_3_O_10_(OH)_2_, 4.17%). The diffraction peaks of the mineral (Quartz) was consistent with the reference library JCP-01-083-0539. The result of the XRD analysis of PQ was in good agreement with the reports by Mishra et al^57^, Torres *et al*^58^, and Amaral *et al*^59^. Similarly, it corroborated the result of the EDX analysis as the elements listed (Fig. 1d) showed consistency with the identified minerals in the diffractogram. The diffractogram of the nanocomposite (Q) identified the same mineral with a reduction in the intensities of the peaks which could be associated with the surface coating and the presence of the carbon nanotubes. However, the diffraction patterns associated with the carbon nanotubes were not observed, which indicated the dominance of the quartzite mineral intensities due to higher crystallinity.

**Figure 3:**
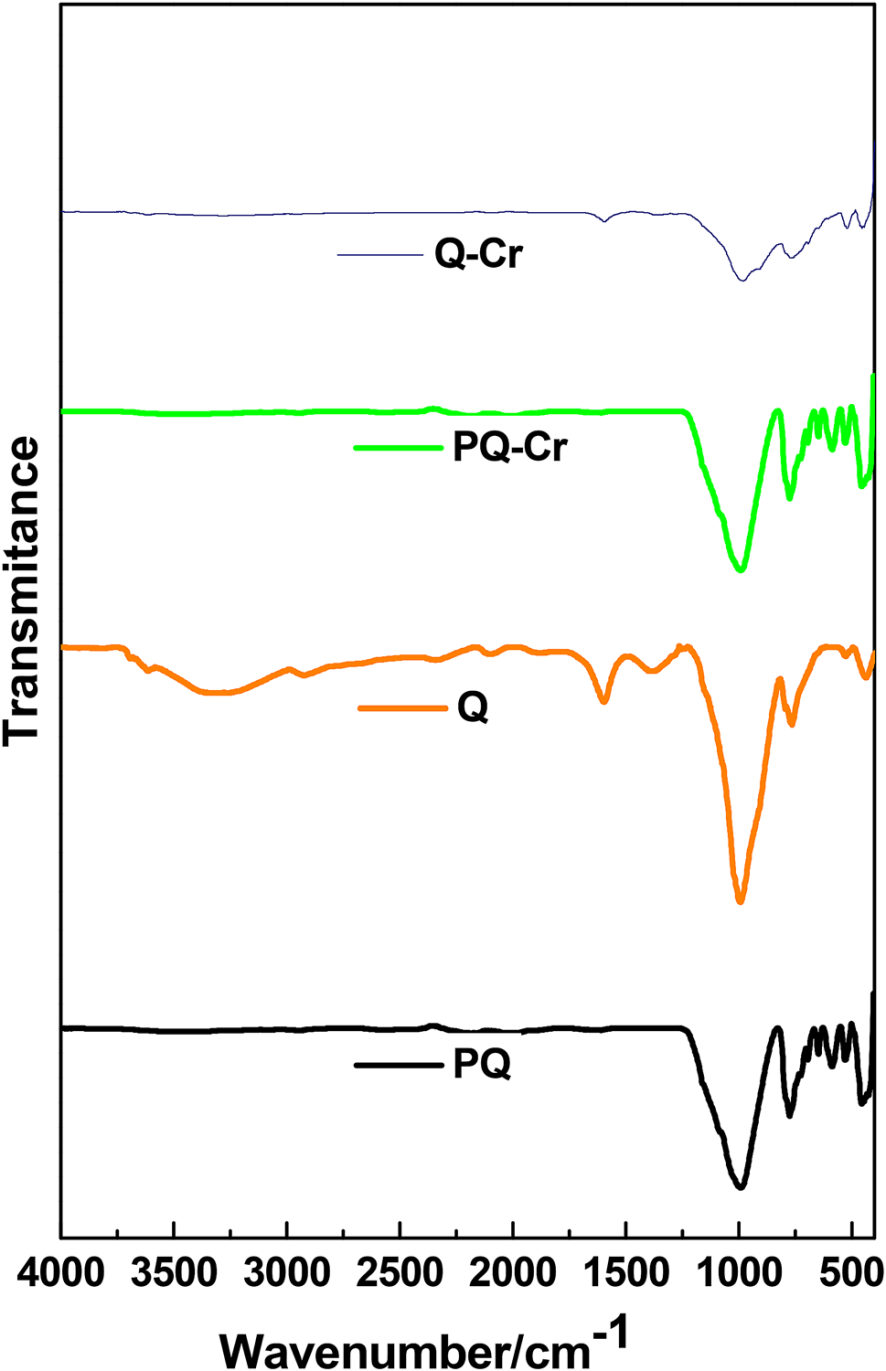
FTIR spectra of PQ, Q, PQ-Cr and Q-Cr.

The FTIR spectra of PQ and Q were recorded in the wavenumber range of 4000–400 cm^−1^. The prominent peaks in the spectrum of PQ (Fig. 3) appeared in the range 1000–400 cm^−1^. These peaks were associated with quartz, which indicated Si-O-Si^60^. However, additional bands were observed in the spectrum of the nanocomposite at (ν/cm^−1^): 1380, 1610, 3500 assigned to asymmetric vibrational stretching modes of carboxylate group (–COO–), the symmetrical C=O stretching of ionic carboxylate groups and the –OH stretching^61–62^. These peaks indicated the incorporation of the *Dacryodes edulis* leaves extract as well as the functionalized carbon nanotubes on the surface of the adsorbent. In a bid to assess the interaction of the adsorbate on the surface of the adsorbents, the FTIR spectra of the loaded adsorbents (PQ-Cr and Q-Cr) were acquired. As shown in Fig. 3, the intensity associated with the band of the hydroxyl functional group on the surface of the nanocomposite was reduced after the adsorption step. This suggested that the -OH functional groups were actively involved in the adsorption of Cr(VI) onto the nanocomposite.

**Figure 4:**
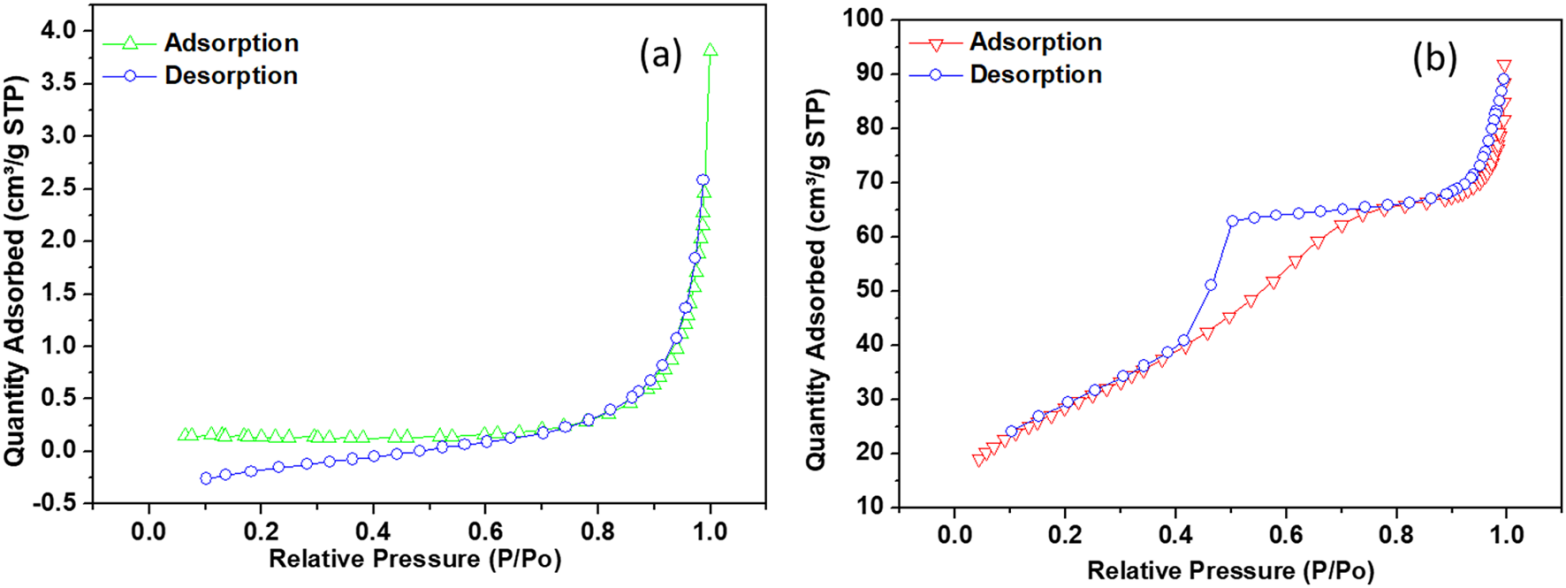
The nitrogen adsorption–desorption curves of (**a**) PQ and (**b**) Q.

The surface area and pore volume of PQ and Q were assessed using the BET nitrogen adsorption–desorption technique. Meanwhile, the pore diameter of PQ and Q were estimated using the Barrett–Joyner–Halenda (BJH) approach. The physisorption isotherm of PQ indicates poor adsorbate-adsorbent interaction. However, the incorporation of carbon nanotubes and plant extract from *Dacryodes* edulis showed a distinct improvement in the specific surface area of the nanocomposite over PQ (Fig. 4). This indicated an increase in the rate of interaction due to the availability of more surface area. Similarly, the increase in pore diameter and pore volume (Table 1) of the nanocomposite (Q) compared to PQ indicated the introduction of macropores along with the existing mesoporous structure of PQ. The form of the graph of amount gas adsorbed versus partial pressure is known as an adsorption isotherm, which are classified as type I–V according to their shape^63^. A type III physisorption isotherm was observed for PQ while a type IV isotherm was observed for the nanocomposite, which indicated the enhanced porosity of the nanocomposite.

**Table 1:** Textural properties of adsorbents.

Adsorbents	Surface area/m^2^ g^−1^	Pore volume/ cm^3^ g^−1^	Pore diameter/ nm	pH_PZC_
PQ	0.3820	0.0061	42.98	4.08
Q	105.6	0.1343	50.90	6.18

**Figure 5:**
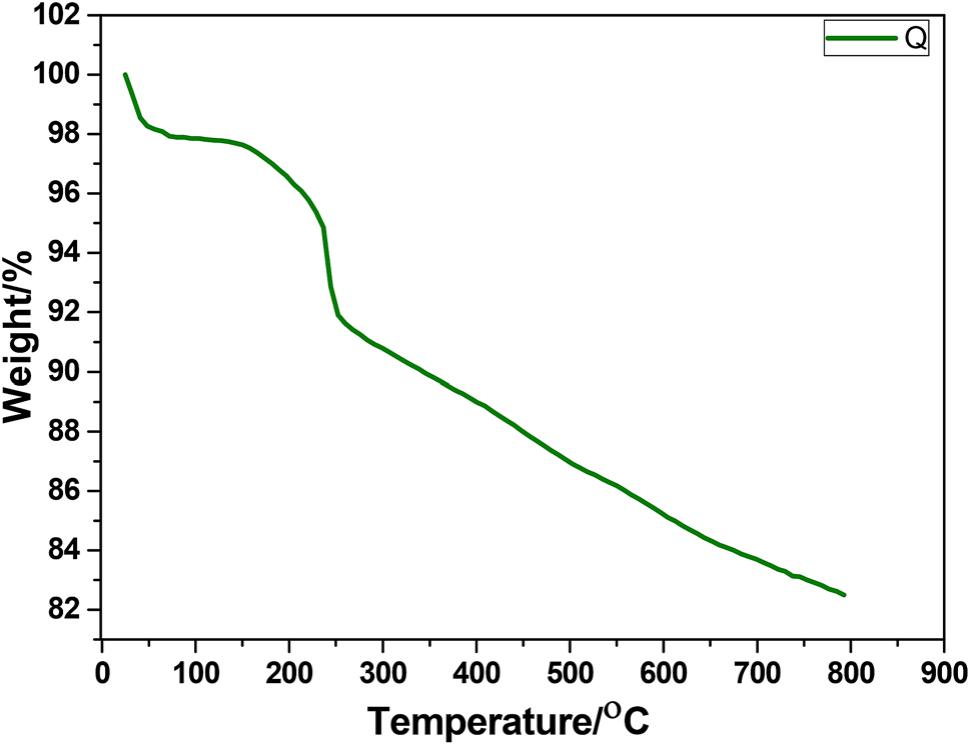
The TGA thermogram of the nanocomposite Q.

The thermal stability of Q was investigated using the thermogravimetric analysis technique. The thermal behavior of pristine quartzite as reported by Xing et al.revealed the loss of physisorbed water only^64^. However, the presence of plant extract and carbon nanotubes strongly influence the thermal stability of the nanocomposite. Figure 5, displayed the thermogram of the nanocomposite which revealed a unique decomposition pattern. About 1.90% mass loss was noticed with the rise of temperature to 110 °C. This could be attributed to the loss of physisorbed water. A further reduction of 2.06% observed as the temperature increased to 300 °C could be associated with the loss of decomposed plant extract on the surface of the nanocomposite and the loss of internally bonded water. However, a progressive decline in mass was observed in the temperature range of 300–800 °C, which reflects the elimination of volatile inorganic or organic materials that were internally bonded. Over the investigated temperature range, about 17.42% mass loss was noticed for the nanocomposite. This accounted for the incorporated plant extract and it showed the stability of quartzite framework, which provided support for the carbon nanotubes used in the fabrication of the nanocomposite. Hence, the fabricated adsorbent can be used to treat wastewater even at high temperatures (< 300 °C) without loss of morphological integrity.

### Effect of pH on Cr(VI) uptake

**Figure 6:**
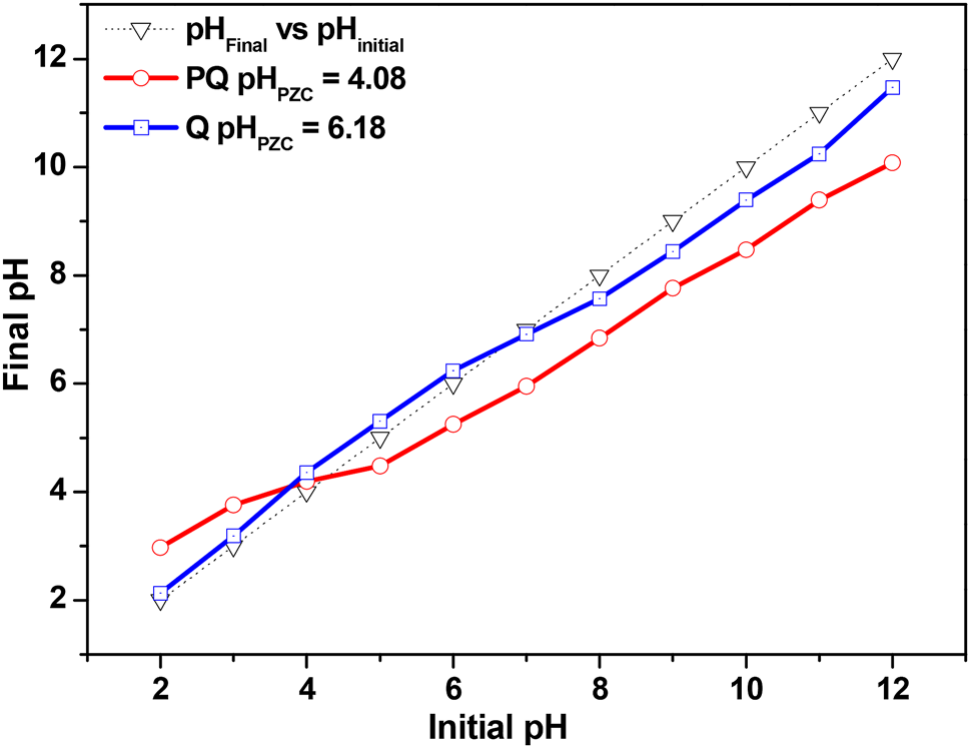
pH_PZC_ plots of PQ and Q.

**Figure 7:**
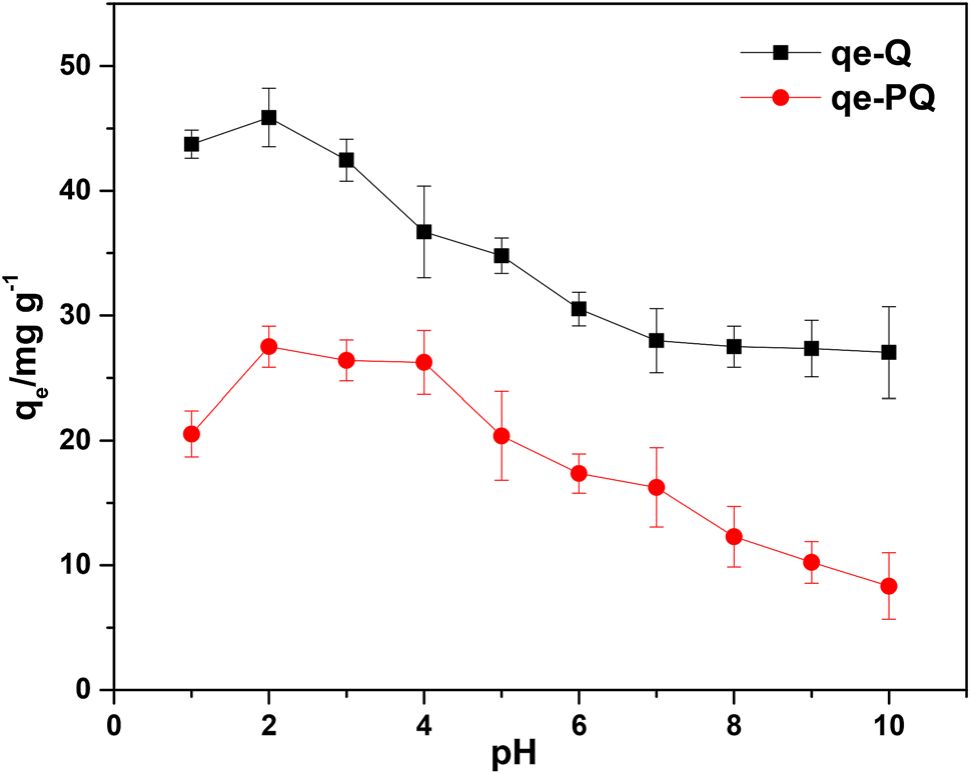
(**a**) The effect of solution pH on the removal capacity of PQ and Q.

Figure 6 revealed the implication of varying sorbate pH on the adsorptive removal of Cr(VI) by PQ and Q. The optimum removal capacities of PQ (27.50 mg g^−1^) and Q (45.87 mg g^−1^) were observed at pH 2. It is noted that at all pH values, the surface modification of PQ to give Q, led to about twice as much removal capacity of Q than for PQ. Meanwhile, the adsorptive capacity of PQ and Q was noticed to decrease with decreased acidity of Cr(VI) solution. Hence, the nanocomposite (Q) demonstrated an adequate potential to eliminate hazardous hexavalent chromium from the aquatic ecosystem. Speciation of hexavalent chromium and the pH_PZC_ of the adsorbents offers better insight on the high Cr(VI) uptake capacity of the sorbent at low solution pH. Note that a reducing argent is necessary to reduce Cr(VI) to Cr(III) at a low pH. As shown in Fig. 7, the pH_PZC_ of PQ and Q were 4.08 and 6.18, respectively. This indicates that at solution pH below and above these values, the surface of the adsorbents (PQ and Q) will be positively and negatively charged respectively. Hence, at solution pH 2, the adsorbents will be positively charged. The hexavalent chromium exists in different oxyanions form with variation in solution pH. In the pH range of 2 to 4, Cr(VI) exist mainly as HCrO_4_^-^ in an aqueous solution. Meanwhile, as the pH increase from 4 to 6, HCrO_4_^−^ is converted to and in equilibrium with Cr_2_O_7_^2–^ ^65–66^. The enhanced uptake capacity of PQ and Q at pH 2 could be due to electrostatic interaction between the positively charged surface of the adsorbents and negatively charged chromium species. It is also possible that Q acts as a reducing agent during the Cr(VI) uptake process, due to the reducing potential of *Dacryodes edulis*^54^. However, finding from this study is in good agreement with the reports from other authors that got optimum pH of 2^22,67^.

### Effect of contact time

**Figure 8:**
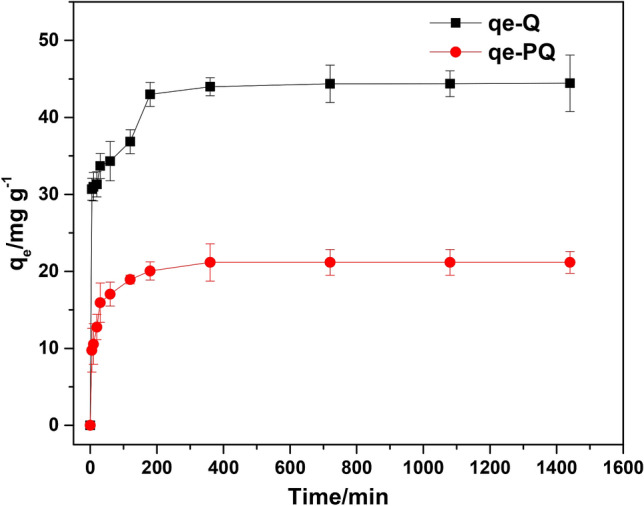
Effect of agitation time on the uptake of Cr(VI) by PQ and Q.

At fixed solution temperature, pH, dosage, and initial Cr(VI) concentration, the effect of contact time on the adsorption of Cr(VI) onto PQ and Q was investigated. As shown in Fig. 8, the time-dependent adsorptive removal of Cr(VI) by PQ and Q was in three stages. The first stage was a fast phase (occurred before 20 min), followed by a gradual increase until 180 min and the last stage that involved no significant increase in the uptake capacity of PQ and Q. Hence, 180 min was selected as the optimum contact time for the elimination Cr(VI) from aqueous solution. However, to ensure complete removal of the adsorbate, 1440 min was employed for further experiment. PQ adsorbed more than double the amount that Q adsorbed at all times > 20 min.

### Kinetics study

**Figure 9:**
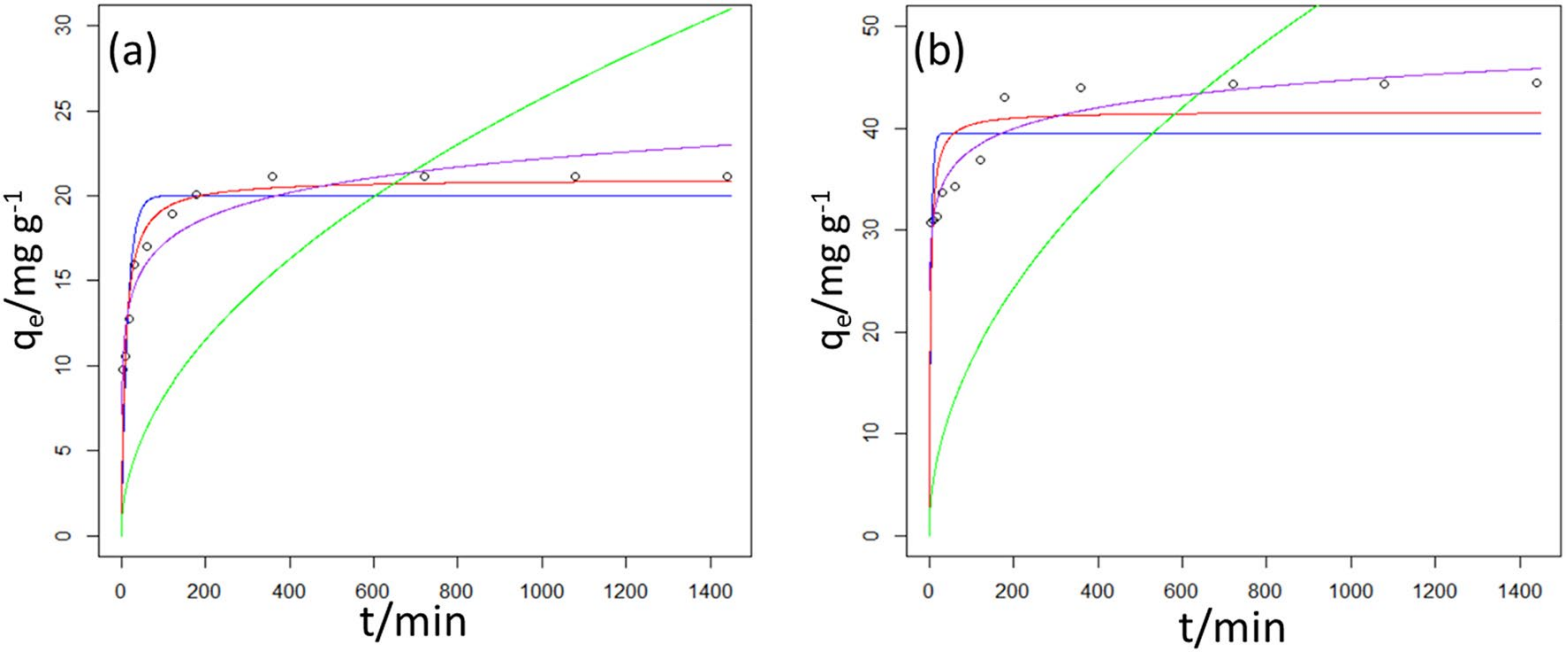
Plots of kinetics models fitted to the experimental data (purple) for the adsorption of Cr(VI) onto (**a**) PQ and (**b**) Q [pseudo-first-order (blue), pseudo-second-order (red), intraparticle diffusion (green), Elovich (black)].

To shed light on the Cr(VI) removal rate by PQ and Q, kinetics model namely pseudo-first-order, pseudo-second-order, Elovich, and Moris-Weber intraparticle diffusion were employed. The nonlinear equations of these models were used to analyze the kinetic data (see Table 2) and the acquired plots are shown in Fig. 9. Meanwhile, the least sum of square residuals (SSR) was used as the goodness-of-fit measure in selecting the model that best describes the data (Table 2). The model with the smallest SSR value gave the best fit to the experimental. The pseudo-first-order kinetic model is best used to describe the adsorption process that is dominantly controlled by diffusion. This model is also based on the fact that an adsorbate binds to a single adsorption site. On the other hand, an adsorption process that is driven by chemisorption is best described by pseudo-second-order kinetic models. In principle, the pseudo-second-order kinetic model assumes binary adsorption of a sorbate. An adsorptive model that demonstrates chemisorption as its rate-determining step is described by the Elovich kinetic model. However, Morris-Weber intraparticle diffusion is used to describe the basic stages involved in the adsorption process. These stages include (i) conveyance of sorbate from the solution bulk to a thin film layer, (ii) transport of sorbate from film layer to the surface of the adsorbent, (iii) migration of sorbate from the adsorbent surface to the interior of the porous structure and (iv) adsorption of sorbate to the adsorption sites. As shown in Table 2, the adsorptive removal of Cr(VI) by PQ and Q was best described by pseudo-second-order (SSR = 10.87) and Elovich kinetic models (SSR = 32.32) respectively. This suggested that chemisorption was the rate-limiting step in the adsorptive removal of hexavalent chromium from the aquatic solution.

**Table 2:** The calculated parameters of the four kinetics models tested for Cr(VI) adsorption onto PQ and Q at different time intervals.

Model	Parameter	P-Q	Q
Experimental	q_exp_/mg g^−1^	21.17	44.44
Pseudo first order	K_1_/min^−1^	0.067	0.224
	q_eq_/mg g^−1^	20.01	39.53
	SSR	36.30	266.3
	RSE	2.008	5.440
Pseudo second order	K_2_/g mg^−1^ min^−1^	0.005	0.008
	q_eq_/mg g^−1^	21.00	41.65
	SSR	10.87	142.4
	RSE	1.099	3.978
	H	2.205	13.88
	t_0.5_/min	9.524	3.001
Intraparticle diffusion	K_id_/mg g^−1^ min^−0.5^	0.814	1.716
	l/mg g^−1^	13.03	31.64
	SSR	800.2	4394
	RSE	8.945	20.96
Elovich	α/mg g^−1^ min^−1^	7.118	24.12
	β/g mg^−1^	2.181	2.986
	SSR	17.80	32.32
	RES	1.406	1.895

### Effect of adsorbent dose

**Figure 10:**
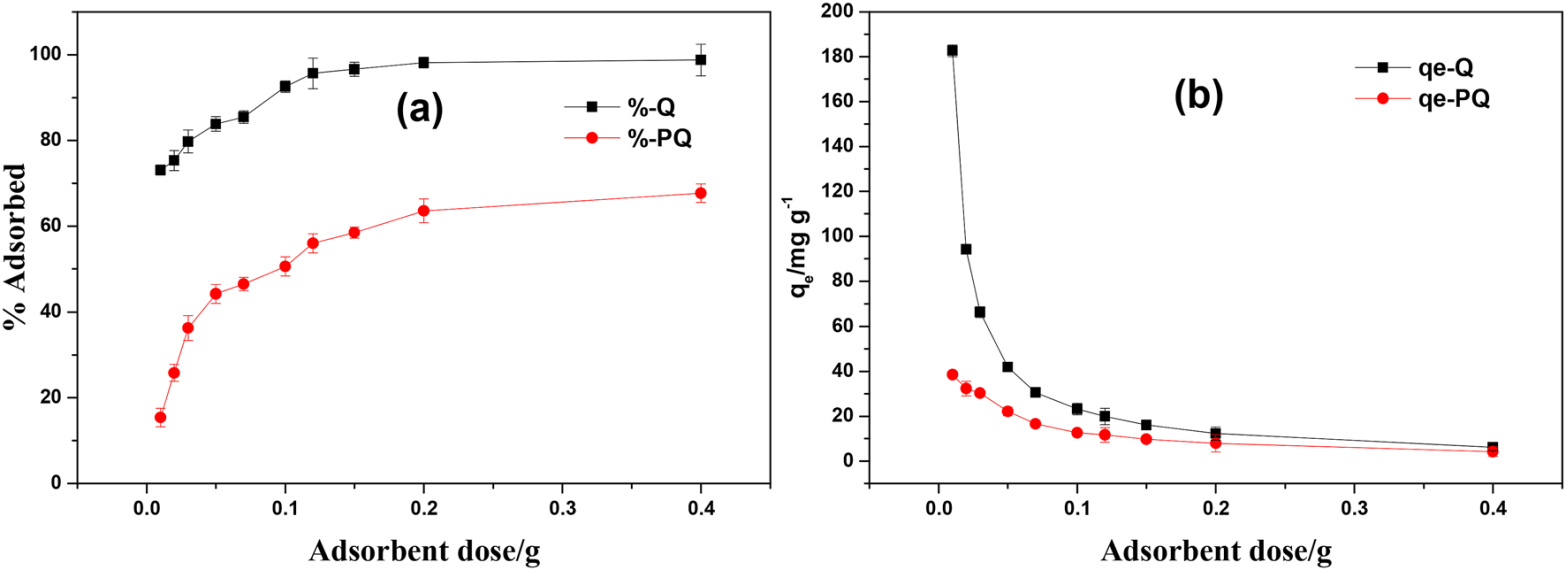
Effect of dosage on adsorption Cr(VI) onto PQ and Q (**a**) % adsorbed and (**b**) adsorption capacity.

As shown in Fig. 10, an increase in removal efficiencies of PQ (15.4 to 67.7%) and Q (73.1 to 98.7%) was noticed as the adsorbent dose was increased from 0.01 to 0.4 g (see Fig. 10). This could be attributed to the increasing amount of adsorption sites at fixed initial Cr(VI) concentration. On the contrary, the uptake capacity of PQ (38.5 to 4.2 mg g^−1^) and Q (182.7 to 6.2 mg g^−1^) were observed to decrease with increased dosage. The decrease in the uptake capacity could be associated with the agglomeration of the sorbent at a higher dosage. Irrespective the adsorbent dose, Q outperformed PQ by having about double the removal capacity of PQ.

### Effect of initial concentration and solution temperature

**Figure 11:**
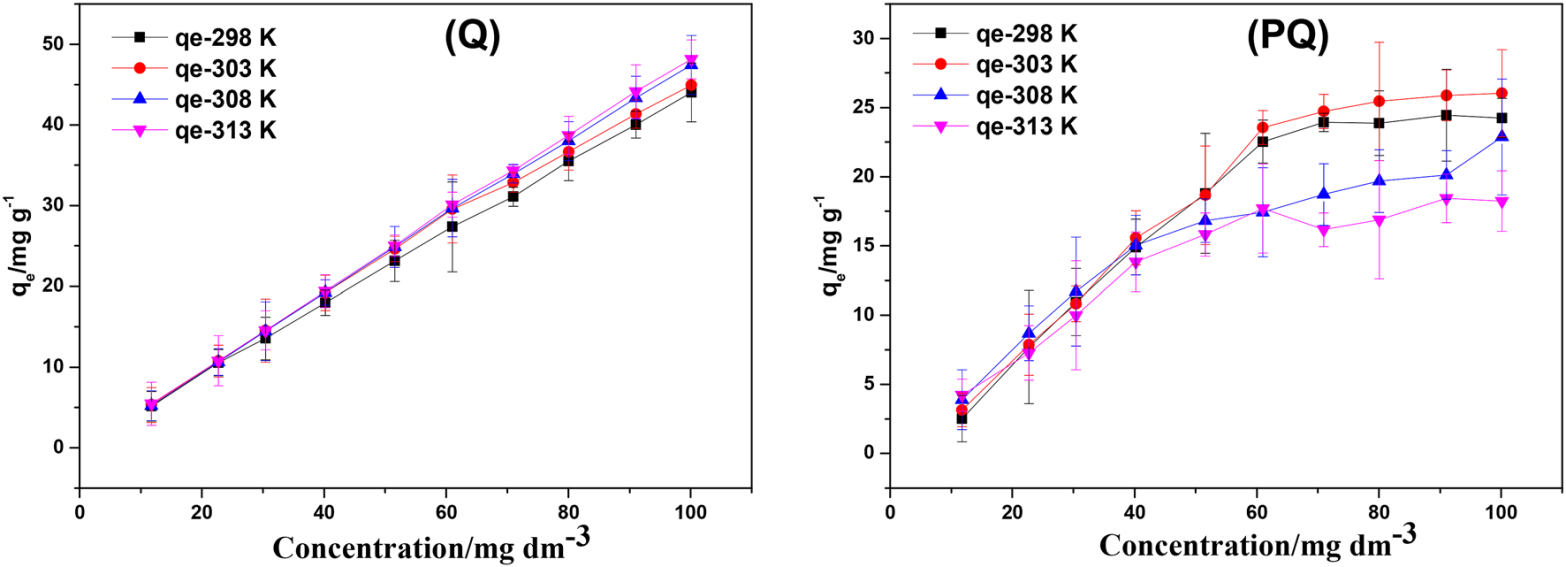
The effect of initial concentration on uptake of Cr(VI) by PQ and Q.

The implication of initial concentration on the adsorptive removal of Cr(VI) by PQ and Q was investigated and the results are displayed in Fig. 11. This study was examined over a concentration range of 10–100 mg g^−1^ with a fixed adsorbent dose of 0.25 g. As shown in Fig. 11, the result revealed that the uptake capacities of PQ and Q increased from 2.5 to 24.3 mg g^−1^ and 5.1 to 44.0 mg g^−1^ respectively as the initial Cr(VI) concentration increased from 10 to 100 mg dm^−3^ at 298 K. It indicated that the adsorptive removal of Cr(VI) onto PQ and Q was strappingly dependent on the initial hexavalent chromium concentration^68^. The phenomenon could be associated with enhanced collisions frequency between the Cr(VI) ions and the adsorption sites on the surface PQ and Q, resulting in higher surface coverage at high initial Cr(VI) concentrations, and thus high uptake capacities. A similar trend was observed at all temperatures investigated. However, an increase in solution temperature was noticed to enhance the uptake capacity of Q, although this effect was more significant at higher solution temperature and thus, demonstration the endothermic adsorptive process. In contrast to this, the influence of solution temperature on the adsorption of hexavalent chromium onto the surface of PQ was trivial. The quantity of Cr(VI) adsorbed at equilibrium, q_e_, is ca double for PQ compared to the Q at the same initial concentration of Cr(VI).

### Adsorption isotherm

The distribution of sorbate in the solid–liquid interphase and the estimation of sorbent potential to eliminate specific sorbate can be determined by making use of mathematical models termed adsorption isotherm. To understand the mechanism of Cr(VI) adsorption onto the surface of PQ and Q, various two- and three-parameter isotherms were used to analyze the equilibrium adsorption data. The least sum of squared residuals (SSR) and the residual squared errors (RSE) were used to select the model that best describes the experimental data. A smaller sum of squared residuals value indicate a better fit of the model to the data; a value of zero means a perfect fit of the model. Among the two-parameter models (Dubinin–Radushkevick, Temkin, Freundlich, Langmuir), the Langmuir isotherm was adequate to describe the adsorption of Cr(VI) onto PQ and Q within the studied temperature range observed (see Table 3). This suggested that the adsorption process of Cr(VI) by PQ and Q was mainly monolayer adsorption. However, those of the three-parameter models (Toth, Redlich-Peterson, Khan, Sips), Sips and R-P were noticed to best fit PQ and Q respectively.

**Table 3:** Isotherm parameters for the adsorption of Cr(VI) onto PQ and Q.

Adsorbent	Isotherm	Parameters	295 K	303 K	310 K	318 K
Q	Langmuir	q_max_	192.5	66.11	387.0	1142
		b	0.024	0.202	0.028	0.013
		SSR	24.44	97.89	161.8	179.5
		RSE	1.748	3.498	4.497	4.737
	Sip	qm	40.03	40.03	49.34	49.34
		b	177.0	177.0	0.118	0.118
		n	1.770	1.770	0.371	0.371
		SSR	3799	3535	85.16	674.39
		RSE	-	-	3.488	-
PQ	Langmuir	qm	36.40	39.25	25.13	22.04
		b	0.056	0.057	0.101	0.092
		SSR	118.7	110.2	23.60	19.47
		RSE	3.851	3.712	1.718	1.560
	R-P	KRP	1.331	1.331	2.246	2.246
		α	0.000	0.000	0.061	0.061
		β	2.340	2.340	1.093	1.093
		SSR	63.32	75.62	23.01	76.59
		RSE	3.008	-	1.813	

*SSR* sum of squared residuals, *RSE* the residual squared errors.

### Comparison of adsorbents for Cr(VI)

A comparison of the maximum monolayer capacity (q_max_) of PQ and Q, with previously reported adsorbents, showed that the nanocomposite reported in this study possess a better absorbance capacity for application in environmental remediation practice (see Table 4). Especially is noted that the surface modification of PQ led to about five times the maximum removal capacity for Q, compared to PQ.

**Table 4:** Comparison of PQ and Q adsorbent with selected related reported works.

Adsorbents	Dosage	pH	q_max_/mg g^−1^	References
Activated carbon	0.1 g	6.0	3.114	^69^
Sugar beet pulp-iron (III) hydroxide	0.5 g	4.4	6.173	^70^
Olive bagasse	0.5 g	2.0	88.59	^71^
Activated red mud	0.5 g	2.0	0.03	^72^
Modified chitosan	0.16 g	5.5	8.310	^73^
Rice straw	10.0 g/L	2.0	3.150	^74^
Functionalized MWCNTs	1.0 g/L	2.85	3.040	^24^
Acrylonitrile grafted banana peels	4.0 g/L	3.0	6.173	^2^
CT-Fe 10%	10 mg	5.5	175.0	^75^
Silica	1.0 g/L	5.0	87.50	^76^
PQ	0.25 g	2.0	36.40	This study
Q	0.25 g	2.0	192.5	This study

### Adsorption thermodynamics

Evaluation of the thermodynamics of adsorption was performed as decribed in the supplementary information. Table 5 revealed that at all temperatures investigated, the ∆G° values of the adsorption of Cr(VI) onto PQ and Q were negative. It showed that the uptake of Cr(VI) by PQ and Q were feasible and spontaneous. Meanwhile, a slight increase in the ∆G° values was noticed with increased solution temperature. This indicated that the removal efficiency of the adsorbents was favored at higher solution temperatures. Furthermore, positive values of ΔH° and ΔS° were estimated from the thermodynamic analysis. This indicated an endothermic adsorption process and an increased haphazardness at the sorbate-sorbent interface respectively. It is worth mentioning that the thermodynamic of an adsorption process creates a path to unveiling the adsorptive mechanism. However, literature revealed that the adsorption process with ΔH° values between 2.1 and 20.9 kJ mol^−1^ is physisorption^77–78^, while ΔH° values are between 80 to 200 kJ mol^−1^ is chemisorption driven^77^. Hence, regarding the ΔH° values of PQ and Q as display in Table 4, the adsorptive removal of Cr(VI) by PQ was a physisorption process while the uptake of Cr(VI) onto Q was a physicochemical process^1^.

**Table 5:** Thermodynamic parameters for the adsorption of Cr(VI) onto PQ and Q.

Adsorbents	T/K	ΔG°/kJ mol^−1^	ΔH/ kJ mol^−1^	ΔS/J K^−1^ mol^−1^
Q	298	− 20.6895		
	303	− 23.9320	34.64	189.7
	310	− 23.9298		
	318	− 25.3097		
QP	298	− 18.6899		
	303	− 19.4312		
	310	− 20.2054	1.194	64.89
	318	− 20.1331		

### Reusability of PQ and Q

**Figure 12:**
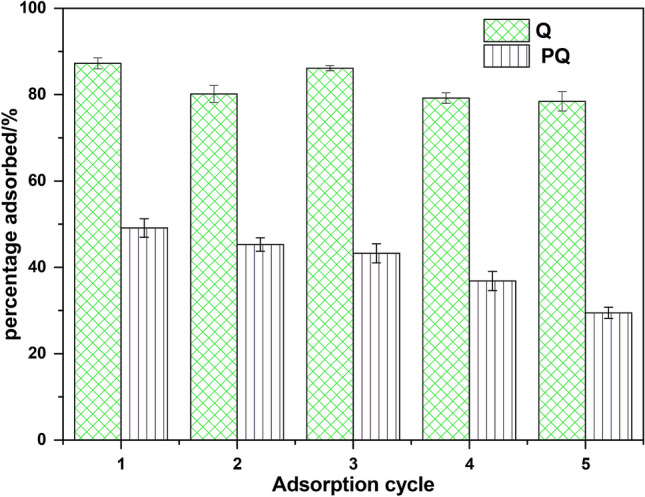
The performance of PQ and Q over five successive adsorption–desorption cycles.

The tendency of PQ and Q to retain their adsorption efficiency after multiple usages were examined and displayed in Fig. 12. To achieve this, the adsorption–desorption cycle was repeated five times. After the fifth cycle, PQ and Q were noticed to have an efficiency of 29% and 78%. The high reusability of Q after 5 cycles is an indicator of the excellent design of Q for Cr(VI) absorbance from waste water. This showed that the enhancing impart of the modifier on the nanocomposite was significant for adsorbent recycling. Hence, Q can be considered to be scaled-up and tested for industrial use.

### Adsorption mechanism

The mechanism responsible for the adsorption of Cr(VI) uptake onto Q may be due to the surface modification of MWCNTs-quartzite using *Dacryodes edulis* leaves extract. The optimum uptake of Cr(VI) onto PQ and Q was established at pH2 (see Fig. 6). Meanwhile, the pH_PZC_ of PQ and Q were 4.08 and 6.18 respectively (see Fig. 7). Hence, at pH2 the positively charged surface of PQ and Q would interact with oxyanions species of Cr(VI) electrostatically. The functional groups of the phytochemical constituent of most plant extract, confer reductive characteristics on extract obtained from different part of plant. [REF]. Hence, the reduction of Cr(VI) to Cr(III) followed by the adsorption of Cr(III) onto the surface of Q via electrostatic interaction may be a possible route for the uptake of Cr(VI). The uptake of Cr(III) could also be via pore entrapment (the enhanced pore volume, pore dimeter and surface area of the nanocomposite (Q) could aid pore entrapment, see Table 1). As shown in Fig. 3, the FTIR spectra of the spent adsorbent (Q-Cr) revealed the disappearance of -OH bands, demonstrating that Cr(VI) chemically interacted with the surface of Q via chemisorption. The pseudo second order model was observed to best describe the uptake of Cr(VI) on to Q (see Table 2), This further justified the inclusion of chemisorption in the uptake of Cr(VI) onto Q.

## Conclusion

A newly fabricated nanocomposite adsorbent (Q) prepared from multiwalled carbon nanotubes (MWCNT) and quartzite (PQ) coated with plant extract was assessed for its capacity to trap/reduce Cr(VI) to its nontoxic species from an aqueous solution. The nanocomposite (Q) showed good surface morphology, undisrupted crystalline phases, enhanced thermal stability, improved pore dimeter and enhanced surface area. Meanwhile, the optimum adsorptive conditions for removal of chromium(VI) by Q was established to be pH 2, adsorbent dose of 50 mg, initial chromium(VI) concentration of 100 mg dm^−3^, the temperature of 318 K, and a contact time of 180 min. Under this conditions Q gave a maximum monolayer capacity (q_max_) of 192.5 mg g^−1^. Furthermore, the experimental isotherms data obtained for the uptake of chromium(VI) onto PQ and Q were best described by the Langmuir model. The time-dependent adsorption data were best described by pseudo-second-order kinetic. The estimated thermodynamic parameters suggested that the removal of chromium(VI) by PQ and Q was spontaneous, endothermic, and entropy-driven. The adsorbent (Q) has demonstrated robust efficiency and large absorbance capacity for the removal of chromium(VI) from aqueous solutions. Owing to the excellent reusability of the adsorbent, Q, giving 78% Cr(VI) absorbance after 5 cycles, Q are recommended to be tested scaled-up conditions for industrial applications.

## Supplementary Information

Supplementary Information.
