# Supplementary material for: Functionalized MWCNTs-quartzite nanocomposite coated with Dacryodes edulis stem bark extract for the attenuation of hexavalent chromium
Source: Sci Rep. 2021 Jun 16;11:12684. doi: 10.1038/s41598-021-92266-0 (PMC8208999; doi:10.1038/s41598-021-92266-0)
Supplement: Supplementary file 1 — Supplementary Information. [file 41598_2021_92266_MOESM1_ESM.docx]

**Functionalized MWCNTs-quartzite nanocomposite coated with *Dacryodes edulis* stem bark extract for the attenuation of hexavalent chromium**

**Amaku James Friday^1*^, Segun A. Ogundare^2^, Kovo G. Akpomie^3,4^, Collins U. Ibeji^4^, Jeanet Conradie^3^**

^1^Department of Chemistry, Michael Okpara University of Agriculture, Umudike, Nigeria

^2^Chemical Sciences Department, Olabisi Onabanjo University, Ago-Iwoye, Nigeria.

^3^Department of Chemistry, University of the Free State, Bloemfontein, South Africa.

^4^Department of Pure & Industrial Chemistry, University of Nigeria, Nsukka, Nigeria.

Corresponding email: [amakufj2006@gmail.com](mailto:amakufj2006@gmail.com) (F. J, Amaku)*

**SUPPLEMENTARY INFORMATION**

**Calculation of adsorption capacity and uptake efficiency**

The adsorption capacities and the uptake efficiency of PQ and Q were calculated by making use of Equations (1) and (2) respectively:

$q_{eq}=\left( \frac{C_{i}-C_{eq}}{m} \right)V$ (1)

$\% adsorbed=\left( \frac{C_{i}-C_{eq}}{C_{i}} \right)\times100$ (2)

Where C_i_ is the initial concentration (mg dm^-3^) of Cr(VI), C_eq_ is the equilibrium concentration (mg dm^-3^), m is the adsorbent mass (g), and V is the volume of Cr(VI) solution (dm^3^).

### Kinetics and isotherm models

The kinetic acquired from time dependent experiment for the adsorption of Cr(VI) onto PQ and Q were used to examine the kinetic process. Four kinetic models, namely, pseudo-first order (Eq. 3) [1], pseudo-second order (Eq. 4) [2], intraparticle diffusion (Eq. 5) [3], and Elovich (Eq. 6) [3, 4] were used to fit the experimental data.

$\frac{{dq}_{t}}{{dt}}=k_{1}\left( q_{e}-q_{t} \right)$ (3)

$\frac{{dq}_{t}}{{dt}_{t}}=k_{2}\left( q_{e}-q_{t} \right)^{2}$ (4)

$\frac{{dq}_{t}}{{dt^{-0.5}}}=k_{id}$ (5)

$\frac{{dq}_{t}}{{dt}_{t}}=\alpha exp\left( -\beta q_{t} \right)$ (6)

Where k_1_, is the pseudo-first-order rate constant (min^-1^); k_2_, represents the pseudo-second-order rate constant (g mg^-1^ min^-1^). k_id_, depicts the intraparticle diffusion rate constant (mg g^-1^ min^0.5^); q_t_, corresponds to the quantity of adsorbate adsorbed at time t (mg g^-1^). Also, q_e_, represents the quantity of adsorbate adsorbed at equilibrium (mg g^-1^); α, is the adsorption rate constant (mg g^-1^ min^-1^), while β, is the desorption rate constant (g mg^-1^).

To establish the maximum adsorption capacity of PQ and Q, the relationship between the uptake capacity of the adsorbents (PQ or Q) and the equilibrium concentration of Cr(VI) was examined. Eight isotherm models, namely, Langmuir (Eq. 7) [5], Freundlich (Eq. 8) [6], Temkin (Eq. 9) [7], Dubinin–Radushkevick (Eq. 10) [8], Sips (Eq. 11) [9], Toth (Eq. 12) [10], Redlich–Peterson (Eq. 13) [11] and Khan (Eq. 14) [12] were employed.

$q_{e}=\frac{q_{max}{bC}_{e}}{1+bC_{e}}$ (7)

$q_{e}=K_{F}C_{e}^{\frac{1}{n}}$ (8)

$q_{e}=\frac{RT}{b_{T}}\ln\left( A_{T}C_{e} \right)$ (9)

$q_{e}=q_{max}e^{{-\beta\varepsilon}^{2}}, \varepsilon=RT\ln\left( 1+\frac{1}{C_{e}} \right)$ (10)

$q_{e}=\frac{{bq}_{max}C_{e}^{\frac{1}{n}}}{1+{bC}_{e}^{\frac{1}{n}}}$ (11)

$q_{e}=\frac{q_{max}C_{e}}{\left( \frac{1}{K_{T}}+C_{e}^{n_{T}} \right)^{\frac{1}{n_{T}}}}$ (12)

$q_{e}=\frac{K_{RP}C_{e}}{1+a_{RP}C_{e}^{g}}$ (13)

$q_{e}=\frac{q_{max}b_{K}C_{e}}{\left( 1+b_{k}C_{e} \right)^{a_{k}}}$ (14)

where q_eq_, adsorption capacity (mg g^-1^) of the adsorbents; C_e_, equilibrium concentration of Cr(VI) in solution (mg dm^-3^); q_max_, maximum monolayer capacity (mg g^-1^); b, Langmuir isotherm constant (dm^3^ mg^-1^); K_F_, Freundlich isotherm constant (mg g^-1^) (dm^-3^ mg^-1^); n, adsorption intensity; b_T_, Temkin isotherm constant; A_T_, Temkin isotherm equilibrium binding constant (dm^-3^ g^-1^); *β*, Dubinin–Radushkevich isotherm constant (mol^2^ kJ^-2^); K_T_, Toth isotherm constant (mg g^-1^); n_T_, Toth isotherm constant; K_RP_, Redlich–Peterson isotherm constant (dm^-3^ g^-1^); a_RP_, Redlich–Peterson isotherm constant; g, Redlich–Peterson isotherm exponent; a_K_, Khan isotherm exponent; b_K_, Khan isotherm constant.

## Adsorption thermodynamics

The feasibility of an adsorption process at a specific temperature can be assessed from cardinal thermodynamic parameters such as enthalpy change (ΔH°), entropy change (ΔS°), and Gibbs energy change (ΔG°). These parameters were estimated from Equations 15 and 16 [13].

$\Delta G^{\circ}=-RT\ln K$ (15)

$\ln K=-\frac{\Delta H^{\circ}}{RT}+\frac{\Delta S^{\circ}}{R}$ (16)

where *R* is the universal gas constant (8.314 J mol^-1^ K^-1^), T is the temperature in Kelvin, *K* was estimated from Langmuir isotherm parameters, the constant is dimensionless and is obtained from K = *q_max_* x *b* x 1000 [14].

**Reference**

1. Aksu, Z. and G. Karabayır, *Comparison of biosorption properties of different kinds of fungi for the removal of Gryfalan Black RL metal-complex dye.* Bioresource Technology, 2008. **99**(16): p. 7730-7741.

2. Sevim, A.M., et al., *An investigation of the kinetics and thermodynamics of the adsorption of a cationic cobalt porphyrazine onto sepiolite.* Dyes and Pigments, 2011. **88**(1): p. 25-38.

3. Ofomaja, A., E. Naidoo, and S. Modise, *Removal of copper (II) from aqueous solution by pine and base modified pine cone powder as biosorbent.* Journal of Hazardous Materials, 2009. **168**(2): p. 909-917.

4. Omorogie, M.O., et al., *Clean technology approach for the competitive binding of toxic metal ions onto MnO2 nano-bioextractant.* Clean Technologies and Environmental Policy, 2016. **18**(1): p. 171-184.

5. Langmuir, I., *The adsorption of gases on plane surface of glass, mica and platinum.* Journal of the American Chemical Society, 1918. **40**(9): p. 1361-1403.

6. Freundlich, H., *Over the adsorption in solution.* Journal of Physical Chemistry, 1906. **57**(385): p. e470.

7. Temkin, M. and V. Pyzhev, *Kinetics of the synthesis of ammonia on promoted iron catalyst.* Journal of Physical Chemistry (USSR), 1939. **13**: p. 851-867.

8. Dubinin, M., *The potential theory of adsorption of gases and vapors for adsorbents with energetically nonuniform surfaces.* Chemical Reviews, 1960. **60**(2): p. 235-241.

9. Sips, R., *On the structure of a catalyst surface.* The Journal of Chemical Physics, 1948. **16**(5): p. 490-495.

10. Toth, J., *State equations of the solid-gas interface layers.* Acta Chimica Academiae Scientiarum Hungaricae, 1971. **69**(3): p. 311-328.

11. Redlich, O. and D.L. Peterson, *A useful adsorption isotherm.* Journal of Physical Chemistry, 1959. **63**(6): p. 1024-1024.

12. Khan, A., I. Al-Waheab, and A. Al-Haddad, *A generalized equation for adsorption isotherms for multi-component organic pollutants in dilute aqueous solution.* Environmental Technology, 1996. **17**(1): p. 13-23.

13. Ho, Y.-S. and A.E. Ofomaja, *Biosorption thermodynamics of cadmium on coconut copra meal as biosorbent.* Biochemical Engineering Journal, 2006. **30**(2): p. 117-123.

14. Milonjić, S.K., *A consideration of the correct calculation of thermodynamic parameters of adsorption.* Journal of the Serbian Chemical Society, 2007. **72**(12): p. 1363-1367.
